# A simpler noninvasive method of predicting markedly elevated pulmonary vascular resistance in patients with chronic thromboembolic pulmonary hypertension

**DOI:** 10.1002/pul2.12102

**Published:** 2022-07-01

**Authors:** Ya‐Nan Zhai, Ai‐Li Li, Xin‐Cao Tao, Wan‐Mu Xie, Qian Gao, Yu Zhang, Ai‐Hong Chen, Jie‐Ping Lei, Zhen‐Guo Zhai

**Affiliations:** ^1^ Department of Cardiology China‐Japan Friendship Hospital Beijing China; ^2^ Institute of Respiratory Medicine Chinese Academy of Medical Sciences Beijing China; ^3^ National Clinical Research Center for Respiratory Diseases Beijing China; ^4^ Department of Pulmonary and Critical Care Medicine China‐Japan Friendship Hospital Beijing China; ^5^ Data and Project Management Unit, China‐Japan Friendship Hospital Institute of Clinical Medical Sciences Beijing China

**Keywords:** chronic thromboembolic pulmonary hypertension, echocardiography, pulmonary endarterectom, pulmonary vascular resistance

## Abstract

Several echocardiographic methods to estimate pulmonary vascular resistance (PVR) have been proposed. So far, most studies have focused on relatively low PVR in patients with a nonspecific type of pulmonary hypertension. We aimed to clarify the clinical usefulness of a new echocardiographic index for evaluating markedly elevated PVR in chronic thromboembolic pulmonary hypertension (CTEPH). We studied 127 CTEPH patients. We estimated the systolic and mean pulmonary artery pressure using echocardiography (sPAP_Echo_, mPAP_Echo_) and measured the left ventricular internal diameter at end diastole (LVIDd). sPAP_Echo_/LVIDd and mPAP_Echo_/LVIDd were then correlated with invasive PVR. Using receiver operating characteristic curve analysis, a cutoff value for the index was generated to identify patients with PVR > 1000 dyn·s·cm^−5^. We analyzed pre‐ and postoperative hemodynamics and echocardiographic data in 49 patients who underwent pulmonary endarterectomy (PEA). In this study, mPAP_Echo_/LVIDd moderately correlated with PVR (*r* = 0.51, *p* < 0.0001). There was a better correlation between PVR and sPAP_Echo_/LVIDd (*r* = 0.61, *p* < 0.0001). sPAP_Echo_/LVIDd ≥ 1.94 had an 77.1% sensitivity and 75.4% specificity to determine PVR > 1000 dyn·s·cm^−5^ (area under curve = 0.804, *p* < 0.0001, 95% confidence interval [CI], 0.66–0.90). DeLong's method showed there was a statistically significant difference between sPAP_Echo_/LVIDd with tricuspid regurgitation velocity^2^/velocity–time integral of the right ventricular outflow tract (difference between areas 0.14, 95% CI, 0.00–0.27). The sPAP_Echo_/LVIDd and mPAP_Echo_/LVIDd significantly decreased after PEA (both *p* < 0.0001). The sPAP_Echo_/LVIDd and mPAP_Echo_/LVIDd reduction rate (ΔsPAP_Echo_/LVIDd and ΔmPAP_Echo_/LVIDd) was significantly correlated with PVR reduction rate (ΔPVR), respectively (*r* = 0.58, *p* < 0.01; *r* = 0.69, *p* < 0.05). In conclusion, the index of sPAP_Echo_/LVIDd could be a simpler and reliable method in estimating CTEPH with markedly elevated PVR and also be a convenient method of estimating PVR both before and after PEA.

## INTRODUCTION

Chronic thromboembolic pulmonary hypertension (CTEPH) is a disease of obstructive pulmonary arteries remodeling as a consequence of major vessel thromboembolism.^1^, ^2^ The occlusion of pulmonary arteries leads to increased pulmonary vascular resistance (PVR) and progressive right heart failure. PVR calculated as the transpulmonary gradient (TPG) divided by pulmonary blood flow is the important parameter of hemodynamic evaluation in the management of CTEPH. Assessment of PVR usually requires right heart catheterization (RHC), which has contraindications and is invasive and unsuitable for continuous evaluation. Noninvasively echocardiographic assessment of PVR is a more accessible method for evaluating CTEPH patients that provides hemodynamics and structural information before and after treatment.

Several echocardiographic methods to estimate PVR have been proposed previously, but none are sufficiently accurate for use in clinical practice. Abbas et al.^3^ found a good correlation between the invasively measured PVR and the ratio of the tricuspid regurgitation velocity (TRV) to the velocity–time integral of the right ventricular outflow tract (TVI_RVOT_). The study did not include patients with PVR over 6 WU. Abbas et al.^4^ demonstrated a more robust association between PVR and TRV^2^/TVI_RVOT_, including patients with PVR > 6 WU. However, the study consisted of only 20 patients with pulmonary hypertension (PH) and only 24 patients with PVR > 6 WU. A further study by Haddad et al.^5^ took into account the contribution of mean right atrial pressure (RAP) and heart rate. We noticed that the above study consisted of patients with various cardiac and pulmonary pathologies and relatively low PVR, and has not been validated in CTEPH.

Chronic right ventricular (RV) pressure overload causes RV enlargement with resultant leftward interventricular septum displacement and decreased left ventricular (LV) preload. The resulting LV dimension and diastolic function reduced has been described in CTEPH.^6^, ^7^ PVR increased in patients with reduced pulmonary blood flow, which will further lead to the reduction of the LV cavity. Therefore, we use echocardiographic estimating systolic pulmonary artery pressure (sPAP_Echo_) or mean pulmonary artery pressure (mPAP_Echo_) and left ventricular internal diameter at end‐diastole (LVIDd) surrogate for transpulmonary pressure and pulmonary blood flow respectively. Our hospital admits lots of CTEPH patients with high PVR (>1000 dyn·s·cm^−5^) who need to be evaluated for the operability of pulmonary endarterectomy (PEA). We hypothesized that we could establish a simpler and more accurate noninvasive method to estimate PVR in CTEPH.

The main objective of our study was to investigate whether the new simple method sPAP_Echo_/LVIDd and/or mPAP_Echo_/LVIDd would provide a better estimation of PVR in situations of markedly elevated PVR in CTEPH.

## METHODS

### Study population

**Figure 1:**
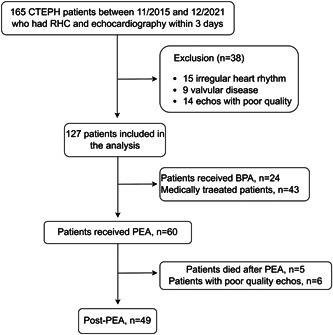
Study flow diagram. BPA, balloon pulmonary angioplasty; CTEPH, chronic thromboembolic pulmonary hypertension; PEA, pulmonary endarterectomy; RHC, right heart catheterization.

We studied consecutive CTEPH patients at our hospital between November 2015 and December 2021. The diagnostic criteria of CTEPH were as follows: PH confirmed by RHC with a pulmonary arterial pressure (PAP; mPAP) ≥25 mmHg and pulmonary arterial wedge pressure ≤ 15 mmHg at rest; ventilation/perfusion imaging had at least one large perfusion defect in one segment or two subsegments; or the evidence of pulmonary vascular lesions identified by computed tomography, magnetic resonance imaging, and/or pulmonary angiography.^1^ Patient with significant left valvular disease (moderate to severe aortic or mitral stenosis or regurgitation), coronary heart disease, cardiomyopathy, irregular heart rhythm, an interval over 3 days between RHC and echocardiography, and/or poor image quality were excluded. In all, 127 CTEPH patients were enrolled in the study. Among these patients, 60 underwent PEA, 24 received balloon pulmonary angioplasty, and 43 were treated with the targeted drug alone. Hemodynamics were measured immediately after surgery and follow‐up echocardiography was performed before hospital discharge in 49 patients who underwent PEA (Figure 1).

### Right heart catheterization

A 7F Swan‐Ganz catheter Philips Allura X‐PER FD20 flat‐plate angiography system (Baxter Inc.) was used to measure systolic, diastolic, and mPAP, mean RAP, and mean pulmonary capillary wedge pressure (PCWP). Cardiac output was measured using the Fick method, which calculated the cardiac index. The TPG was calculated by subtracting the mean PAP from the PCWP. PVR (dyn·s·cm^−5^) was calculated by dividing the TPG by the cardiac output. Of those patients that underwent PEA, pulmonary artery pressure and PVR were measured immediately after surgery.

### Echocardiography

**Figure 2:**
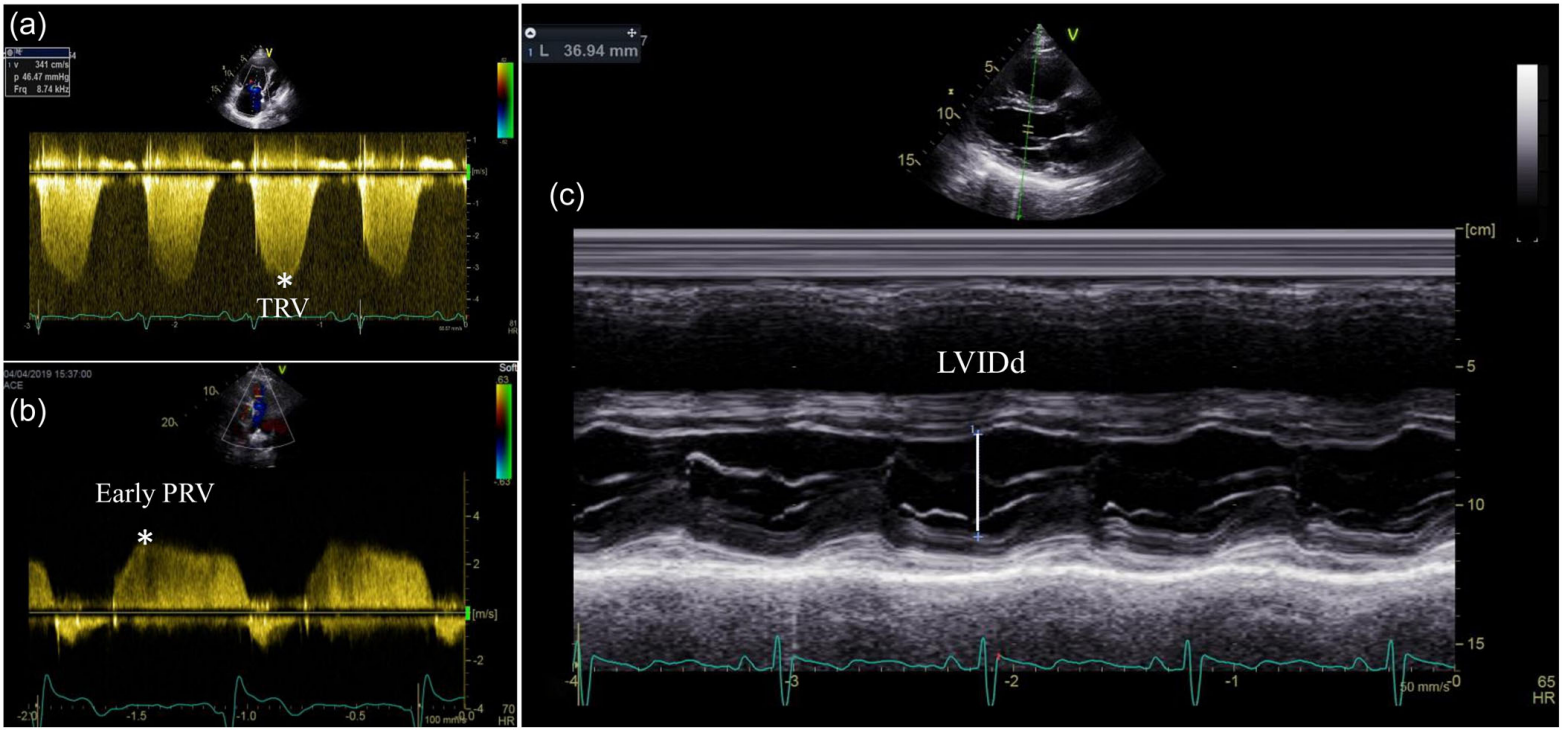
(a) The tricuspid regurgitation velocity (TRV) was measured to calculate systolic pulmonary artery pressure. (b) The early diastolic pulmonary regurgitation velocity (PRV) was measured to calculate mean pulmonary artery pressure. (c) Left ventricular internal diameter at end‐diastole (LVIDd) was acquired in the parasternal long‐axis view at a level of the mitral leaflet tips by M‐mode.

A comprehensive echocardiographic examination was performed within 3 days of RHC before PEA and 14 ± 4 days (range 2–32 days) after PEA by experienced dedicated cardiologists (Ai‐li and Ya‐Nan), using the Vivid E95 ultrasound system (General Electric Healthcare, Vingmed) with an M5S transducer. The subjects were placed in the left lateral position, and at least three consecutive beats were stored. Two‐dimensional and Doppler echocardiography were performed in accordance with current guidelines.^8^ Analysis of the images was performed offline using the EchoPac software version 201 (General Electric Healthcare, Vingmed). LVIDd was acquired in the parasternal long‐axis view by M‐mode, and an electrocardiogram was used to determine the position of the end‐diastole accurately. The ultrasound beam is aligned so that it is perpendicular to the interventricular septum and posterior wall at a level of the mitral leaflet tips. TVI_RVOT_ was obtained by placing a 1–2‐mm pulsed‐wave Doppler sample volume in the proximal RV outflow tract when imaged from the parasternal short‐axis view. The average of three measurements was used for LVIDd and TVI_RVOT_. The peak TRV was measured as the highest of the velocities obtained from the lower parasternal and apical multiple views. The early diastolic pulmonary regurgitation velocity (PRV) was measured as the highest of the velocities obtained from the parasternal short‐axis view (Figure 2).

Noninvasive estimation of RAP was based on the size and collapse index of the inferior vena cava(IVC) and was scored as either 3, 8, or 15 mmHg.^8^ Doppler sPAP_echo_ was estimated as 4 × TRV^2^ + estimated RAP. Doppler mPAP_echo_ was estimated as 4 × (early PRV)^2^ + estimated RAP.

The first method was based on the index of sPAP_Echo_/LVIDd. The second method was based on the ratio of mPAP_Echo_/LVIDd. The TRV^2^/TVI_RVOT_ ratio was also calculated, as described by Abbas et al.^4^

### Statistical analysis

Continuous variables were expressed as mean ± SD or median (interquartile range) and compared using the *t*‐test or Mann–Whitney *U* test, as appropriate. Categorical variables were expressed as percentages and compared using the Pearson *χ* ^2^ test or Fisher's exact test, as appropriate. Correlations between variables were assessed using Spearman correlation tests. Receiver operating characteristics (ROC) curves were performed to determine echocardiographic cutoff values for detecting PVR > 1000 dyn·s·cm^−5^. Comparison of area under curve (AUC) between groups was performed using the DeLong method. Paired *t*‐test and Wilcoxon signed rank test was used for pre‐ and post‐PEA comparisons. Intraobserver and interobserver reproducibility were assessed in randomly selected 30 subjects. Interobserver reproducibility was tested by two independent observers. Interobserver and intraobserver reproducibility were evaluated by means of intraclass correlation coefficient (ICC). Statistical analyses were performed using SPSS 17.0 software (SPSS Inc.).

## RESULTS

### Patient characteristics

The main clinical, hemodynamic, and basic echocardiographic characteristics of the studied patients are presented in Table 1. The mean mPAP was 43.7 ± 10.8 mmHg, the mean RAP was 3 (1–6) mmHg, the mean confidence interval (CI) was 2.2 ± 3.7 L/min/m^2^, the mean PVR was 987.2 ± 483.1 dyn·s·cm^−5^. Fifty‐two of 127 patients (40.9%) had a PVR > 1000 dyn·s·cm^−5^. TRV and sPAP_Echo_ could be obtained in 113 of 127 patients (89.0%). PRV and mPAP_Echo_ could be obtained in 74 of 127 patients (58.3%).

**Table 1:** Baseline characteristics of patients with CTEPH

Demographic characteristics	All patients (*n* = 127)
Age (year)	57.5 (49–63)
Men (%)	68 (53.5%)
Heart rate (bpm)	77.2 ± 12.2
BSA (m^2^)	1.72 (1.6–1.87)
WHO functional class	
I/II	69 (54.8)
III/IV	58 (45.6)
6MWD (mm)	390 (290–450)
NT‐proBNP (pg/mL)	651 (209–1387)
Right heart catheterization	
Systolic PAP (mmHg)	79.3 ± 19.8
Mean PAP (mmHg)	43.7 ± 10.8
Mean RAP (mmHg)	3 (1‐6)
PAWP (mmHg)	8.4 ± 3.8
PVR (dyn·s·cm^−5^)	987.2 ± 483.1
CI (L/min/m^2^)	2.2 ± 3.7
SvO_2_ (%)	64.8 ± 9.1
Echocardiography	
LVEF (%)	68.9 ± 4.6
LVIDd (mm)	41.5 ± 5.2
RA minor‐axis dimension (mm)	50.6 ± 10.4
RV basal diameter (mm)	46.6 ± 6.8
RV/LV basal diameter ratio	1.2 (1.1–1.5)
TRV (m/s)	4.2 ± 5.8
sPAP_Echo_ (mmHg)	80.3 ± 20.8
PRV (m/s)	2.8 ± 3.9
mPAP_Echo_ (mmHg)	39.2 ± 10.6
TVI_RVOT_ (cm)	9.9 (7.8–12.0)
TAPSE (mm)	16.4 ± 3.4
S' (cm/s)	9.8 ± 2.3
RVFAC (%)	29.4 ± 7.5
TRV^2^/TVI_RVOT_	2.1 ± 1.2
mPAP_Echo_/LVIDd	0.98 ± 0.32
sPAP_Echo_/LVIDd	1.99 ± 0.65

Abbreviations: 6MWD, 6‐min walk distance; BSA, body surface area; CI, confidence interval; CTEPH, chronic thromboembolic pulmonary hypertension; LV, left ventricular; LVEF, left ventricular ejection fraction; LVIDd, left ventricular end‐diastolic diameter; mPAP_Echo_, echocardiographic determination of mean PAP; NT‐proBNP, N‐terminal fragment of pro‐B‐type natriuretic peptide; PAH, pulmonary arterial hypertension; PAP, pulmonary artery pressure; PAWP, pulmonary artery wedge pressure; PRV, pulmonary regurgitation velocity; PVR, pulmonary vascular resistance; RA, right atrial; RAP, right atrial pressure; RV, right ventricular; RVFAC, right ventricular fractional area change; sPAP_Echo_, echocardiographic determination of systolic PAP; TAPSE, tricuspid annular plane systolic excursion; TRV, tricuspid regurgitation velocity; TVI_RVOT_, velocity–time integral of the right ventricular outflow tract; WHO, World Health Organization.

#### Linear regression and ROC analysis in CTEPH patients

**Figure 3:**
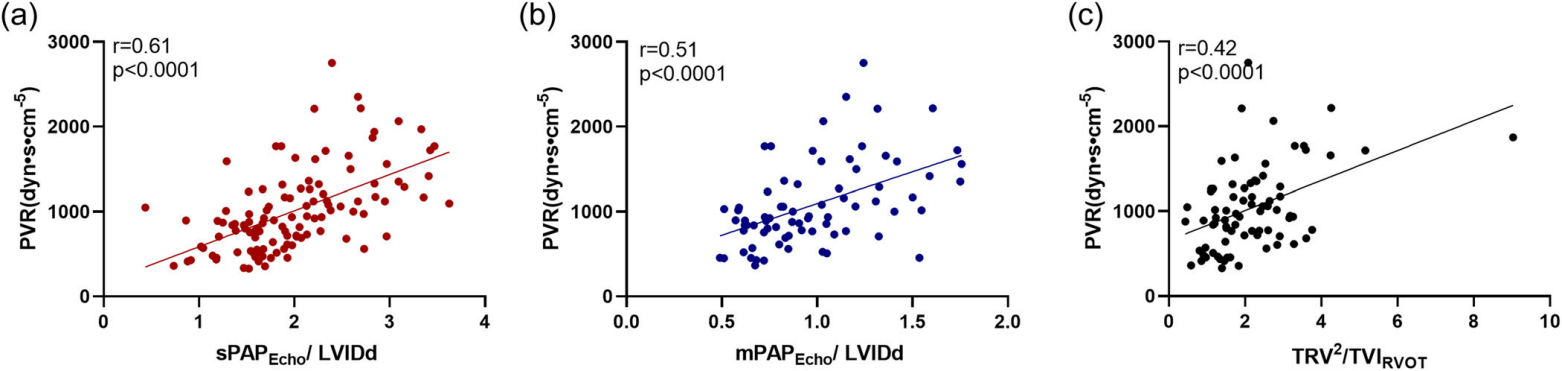
Linear regression analysis between PVR and sPAP_Echo_/LVIDd (a), mPAP_Echo_/LVIDd (b), and TRV^2^/TVI_RVOT_ (c). LVIDd, left ventricular end‐diastolic diameter; mPAP_Echo_, echocardiographic determination of mean PAP; PAP, pulmonary artery pressure; PVR, pulmonary vascular resistance; sPAP_Echo_, echocardiographic determination of systolic PAP; TRV, tricuspid regurgitation velocity; TVI_RVOT_, velocity–time integral of the right ventricular outflow tract.

Linear regression analysis between PVR and TRV^2^/TVI_RVOT_ revealed a weak correlation (*r* = 0.38, *p* < 0.01; Figure 3c). mPAP_Echo_/LVIDd correlated moderately with PVR (*r* = 0.51, *p* < 0.0001; Figure 3b). There was a better correlation between PVR and sPAP_Echo_/LVIDd (*r* = 0.61, *p* < 0.0001; Figure 3a).

**Figure 4:**
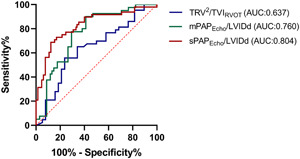
Receiver operating characteristic curves for TRV^2^/TVI_RVOT_, sPAP_Echo/_LVIDd, and mPAP_Echo/_LVIDd to distinguish PVR > 1000 dyn·s·cm^–5^. AUC, area under the curve; LVIDd, left ventricular end‐diastolic diameter; mPAP_Echo_, echocardiographic determination of mean PAP; PAP, pulmonary artery pressure; PVR, pulmonary vascular resistance; sPAP_Echo_, echocardiographic determination of systolic PAP; TRV, tricuspid regurgitation velocity; TVI_RVOT_, velocity–time integral of the right ventricular outflow tract.

Figure 4 represents the ROC curves of mPAP_Echo_/LVIDd, sPAP_Echo_/LVIDd, and the TRV^2^/TVI_RVOT_. mPAP_Echo/_LVIDd ≥ 0.97 had an 70.6% sensitivity and 77.5% specificity to determine PVR > 1000 dyn·s·cm^−5^. Its AUC is 0.760 (*p* < 0.0001, 95% CI, 0.62–0.87). sPAP_Echo/_LVIDd ≥ 1.94 had an 77.1% sensitivity and 75.4% specificity to determine PVR > 1000 dyn·s·cm^−5^. Its AUC is 0.804 (*p* < 0.0001, 95% CI, 0.66–0.90). The AUC for TRV^2^/TVI_RVOT_ was 0.637. sPAP_Echo/_LVIDd had the largest AUC. There was a statistically significant difference between sPAP_Echo/_LVIDd with TRV^2^/TVI_RVOT_ (difference between areas 0.14, 95% CI, 0.00–0.27).

#### Hemodynamic and echocardiography after PEA

Table 2 shows the RHC and echocardiographic characteristics in patients with pre‐ and post‐PEA.

**Table 2:** RHC and echocardiographic characteristics pre‐ and post‐PEA (*n* = 49)

	Pre‐PEA	Post‐PEA	*p*
RHC measurements			
Mean PAP (mmHg)	43.3 ± 11.7	25.5 ± 6.5	<0.001
PVR (dyn·s·cm^−5^)	930.9 ± 506.0	486.4 ± 177.8	<0.001
Echocardiographyic measurements			
LVIDd (mm)	41.1 ± 5.5	44.2 ± 4.2	<0.001
RA minor‐axis dimension (mm)	52.2 ± 11.3	41.5 ± 66.9	<0.001
RV basal diameter (mm)	48.3 ± 6.8	39.4 ± 5.0	<0.001
RV/LV basal diameter ratio	1.30 (1.07–1.60)	1.00 (0.90–1.10)	<0.001
TRV (m/s)	4.3 ± 4.8	3.1 ± 5.9	<0.001
sPAP_Echo_ (mmHg)	82.5 ± 19.7	43.1 ± 15.6	<0.001
PRV (m/s)	2.9 ± 3.8	2.2 ± 3.6	<0.001
mPAP_Echo_ (mmHg)	41.8 ± 11.1	24.2 ± 7.4	<0.001
TVI_RVOT_ (cm)	8.8 ± 2.4	14.5 ± 5.1	<0.01
TAPSE (mm)	16.2 ± 3.5	11.5 ± 3.3	<0.001
S' (cm/s)	9.8 ± 2.2	7.8 ± 1.9	<0.001
RVFAC (%)	28.2 ± 5.1	38.8 ± 8.5	<0.01
TRV^2^/TVI_RVOT_	2.37 ± 1.57	0.67 ± 0.36	<0.01
mPAP_Echo_/LVIDd	1.02 ± 0.36	0.55 ± 0.15	<0.001
sPAP_Echo_/LVIDd	2.04 ± 0.70	0.97 ± 0.32	<0.001

Abbreviations: LV, left ventricular; LVIDd, left ventricular end‐diastolic diameter; mPAP_Echo_, echocardiographic determination of mean PAP; PAP, pulmonary artery pressure; PEA, pulmonary endarterectomy; PRV, pulmonary regurgitation velocity; PVR, pulmonary vascular resistance; RA, right atrial; RHC, right heart catheterization; RV, right ventricular; RVFAC, right ventricular fractional area change; sPAP_Echo_, echocardiographic determination of systolic PAP; TAPSE, tricuspid annular plane systolic excursion; TRV, tricuspid regurgitation velocity; TVI_RVOT_, velocity–time integral of the right ventricular outflow tract.

Directly after PEA, mPAP decreased from 43.3 ± 11.7 to 25.5 ± 6.5 mmHg; PVR from 930.9 ± 506.0 to 486.4 ± 177.8 dyn·s·cm^−5^ (both *p* < 0.0001). Post‐PEA resulted in a significant decrease in RA and RV dimensions and an increase in LVIDd (both *p* < 0.0001). sPAP_Echo_, mPAP_Echo_ significantly decreased after PEA (both *p* < 0.0001). RVFAC significantly increased after PEA (*p* < 0.001). TAPSE and S' decreased significantly in the early postoperative period due to the impaired longitudinal contraction after surgery (*p* < 0.0001).

**Figure 5:**
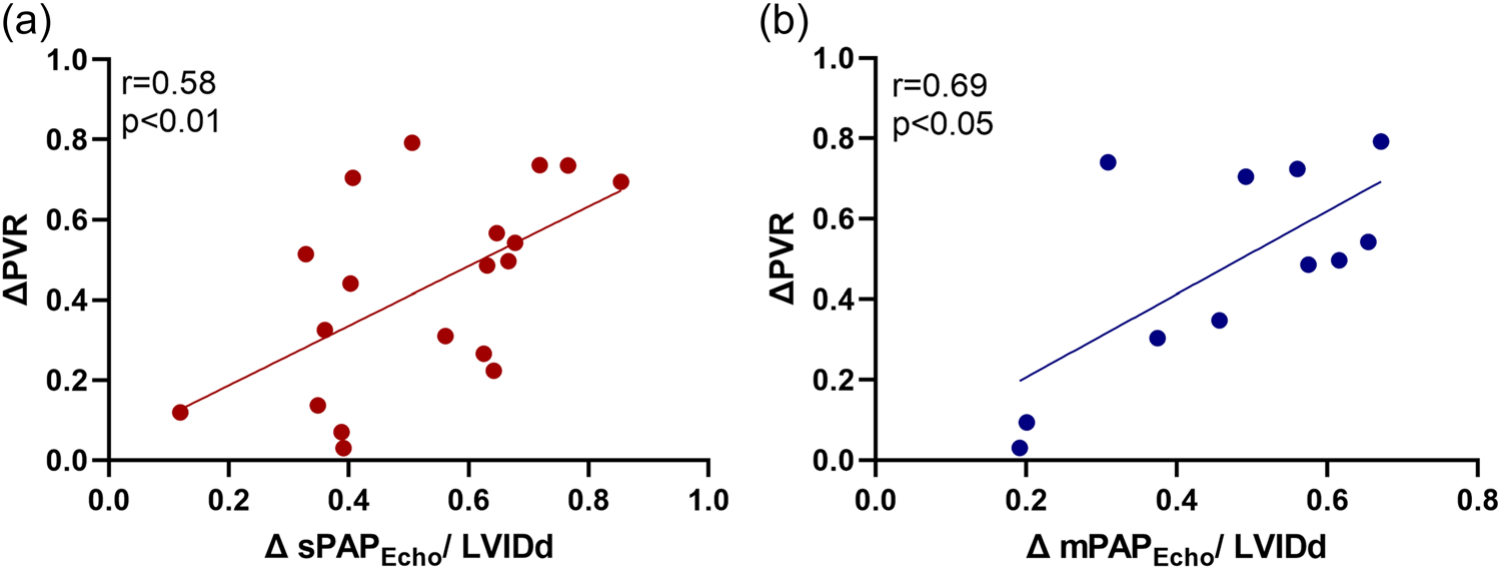
Linear regression analysis between PVR reduction rate (ΔPVR) and sPAP_Echo/_LVIDd reduction rate (ΔsPAP_Echo/_LVIDd) (a) and mPAP_Echo/_LVIDd reduction rate (ΔmPAP_Echo/_LVIDd) (b). LVIDd, left ventricular end‐diastolic diameter; mPAP_Echo_, echocardiographic determination of mean PAP; PAP, pulmonary artery pressure; PVR, pulmonary vascular resistance; sPAP_Echo_, echocardiographic determination of systolic PAP.

The sPAP_Echo/_LVIDd and mPAP_Echo/_LVIDd significantly decreased after PEA (both *p* < 0.0001). The sPAP_Echo/_LVIDd and mPAP_Echo/_LVIDd reduction rate (ΔsPAP_Echo/_LVIDd and ΔmPAP_Echo/_LVIDd) was significantly correlated with PVR reduction rate (ΔPVR), respectively (*r* = 0.58, *p* < 0.01; *r* = 0.69, *p* < 0.05; Figure 5).

### Reproducibility

The interobserver ICC was 0.83 for TVI_RVOT_ and 0.97 for LVIDd, and the intraobserver ICC was 0.88 and 0.99 for TVI_RVOT_ and LVIDd. LVIDd demonstrated better interobserver and intraobserver reproducibility than TVI_RVOT_.

## DISCUSSION

This study demonstrates that the index of sPAP_Echo/_LVIDd represents a simpler and better method of estimating markedly elevated PVR in CTEPH patients.

A number of echocardiographic methods have been proposed for the noninvasive estimation of PVR.^3^, ^4^, ^9^, ^10^, ^11^ The guidelines of the American Society of Echocardiography recommended the noninvasive estimation of PVR should not be used as a substitute for invasive evaluation of PVR.^12^ The application of echocardiography can provide the range of PVR values for the clinical practice rather than calculating PVR values by a formula. PVR is widely acknowledged as a prognostic factor for CTEPH patients. PVR higher than 1000 dyn·s·cm^−5^ is considered to be severe and is correlated with an increased risk of postoperative mortality.^13^, ^14^ We validated previously reported methods and proposed a simple method to help clinically identify CTEPH patients with extremely high PVR values (>1000 dyn·s·cm^−5^) instead of generating a regression equation. We believe that the significance of echocardiographic estimation of PVR in CTEPH patients is to identify patients with high PVR and judge their prognosis and treatment options.

Our results showed that the correlation between PVR with sPAP_Echo/_LVIDd and mPAP_Echo/_LVIDd appeared stronger compared with TRV^2^/TVI_RVOT_. The correlation between PVR and PVR obtained by the formula in our study was weaker compared with previous studies, which may be related to the fact that the subjects of previous studies were mostly patients with relatively low PVR values, while our patients included high PVR and wide distribution of PVR values. Moreover, our results showed the AUC of sPAP_Echo_/LVIDd is higher than the AUC of TRV^2^/TVI_RVOT_ to determine PVR > 1000 dyn·s·cm^−5^. Patients in previous studies were composed of patients with relatively low PVR and various etiologies. The new method may be more applicable to identifying extremely elevated PVR in CTEPH patients.

Compared to the method of Abbas et al.^4^ and Kaga et al.,^10^ the new method also takes into account the contribution of RAP to estimate sPAP and mPAP. Current guidelines have suggested that RAP predicts the prognosis of PAH.^1^ For patients with PVR > 1000 dyn·s·cm^−5^, RAP will also be higher. So, the estimated RAP will contribute more to mPAP_Echo_ in patients with PVR > 1000 dyn·s·cm^−5^. Although Haddad et al.^5^ took RAP into account, according to the size and collapse index of the IVC. RAP was estimated to be 10 mmHg, 15 mmHg, or 20 mmHg, which would lead to an overestimation of RAP.

Echocardiographically, the interventricular septum is often markedly flattened in severe CTEPH, and the LV appears compressed, due to the presence of right ventricular pressure overload. Ehtisham et al. demonstrated that RV overload causes an absolute decrease in early LV filling without late diastolic compensation, corresponding with a low stroke volume and cardiac output in CTEPH.^15^ With removal of the pulmonary artery thrombi and improved CO after PEA, this underfilling is corrected, leading to increase of LV volume, which is supported by our results. Therefore, LV end‐diastolic volume (LVEDV) represents pulmonary blood flow in CTEPH and is significantly reduced in patients with extremely elevated PVR. LVIDd, as an alternative indicator of LVEDV, is easy to obtain and more suitable for routine applications. Moreover, LVIDd had better repeatability and accuracy than TVI_ROVT_. In patients with CTEPH, the dilatation of main pulmonary artery caused pulmonary valve to move forward. When obtaining the flow velocity in the RV outflow tract, the Doppler sampling line had a larger angle with the direction of blood flow, which would affect the accuracy of the measurement of TVI_ROVT_. In addition, when the velocity of the RV outflow tract was low, the contour of the spectrum was not clearly displayed, which would affect the repeatability of the measurement. For patients in the early postoperative period of PEA, some patients in our study received bedside echocardiography or poor image quality and could not obtain satisfactory TVI_ROVT_.

In our study, tricuspid regurgitation (TR) could be obtained in 113 of 127 patients (89.0%). In addition to patients without TR, others did not have well‐defined TR signal quality or had severe TR, which may lead to overestimation or underestimation of PAP. In our study, pulmonary regurgitation (PR) could be obtained in 74 of 127 patients (58.3%). We can measure PRV to estimate mPAP under these circumstances. It has been reported that the early‐diastolic pulmonary artery‐right ventricular pressure gradients derived from the peak early‐diastolic PR velocity are useful for estimating the mPAP.^12^, ^16^, ^17^ PR is reported to occur in almost 75% of the population.^18^ Dilation of the annulus and main pulmonary artery are usually accompanied by CTEPH patients. PR secondary to PH is common. Thus, it is easy to obtain the complete PR Doppler signal. Therefore, we consider that sPAP_Echo_/LVIDd can be used to predict markedly elevated PVR when TR signal quality is acceptable. If the TR signal is incomplete or severe TR exists, mPAP_Echo_/LVIDd can also be used to predict markedly elevated PVR when a high‐quality PR signal is available.

sPAP_Echo_/LVIDd is more reliable in predicting CTEPH with markedly elevated PVR when a high‐quality TR signal is available. The application of the simpler formula in routine echocardiography in CTEPH can predict patients with PVR > 1000 dyn·s·cm^−5^, which is valuable for disease severity and convenient monitoring of PVR before and after treatment. LVIDd instead of transpulmonary blood flow is suitable for patients without left heart disease and severe valve regurgitation. And whether it can extend to other precapillary PH needs further validation.

## LIMITATIONS

There are several limitations to this study. Although the clinical condition of the patients was stable during each examination, RHC and echocardiography were not performed simultaneously, which affected the accuracy of the comparison. Second, we did not include echocardiographic measurement of PCWP, despite the fact that none of the patients had a significant left valvular disease. Third, the sample size of post‐PEA patients was small and some of them had neither TR or PR to obtain sPAP_Echo_/LVIDd or mPAP_Echo_/LVIDd. Finally, this study was a single‐center retrospective study. Further prospective multicenter studies involving larger patient populations are required to confirm the results.

## CONCLUSION

The simpler and noninvasive index, sPAP_Echo_/LVIDd, can be clinically used in the routine echocardiographic evaluation and can be a valuable method of estimating markedly elevated PVR (>1000 dyn·s·cm^−5^) in CTEPH patients. The present study demonstrated that sPAP_Echo_/LVIDd and mPAP_Echo_/LVIDd decrease along with the reduction of PVR immediately after PEA in CTEPH patients, which may be a convenient method of estimating PVR both before and after PEA.

## AUTHOR CONTRIBUTIONS

Ai‐Li Li created the idea and designed the study. Ai‐Li Li and Ya‐Nan Zhai performed research, collected data, analyzed the results, and wrote the manuscript. Xin‐Cao Tao provided the data of RHC. Wan‐Mu Xie and Qian Gao helped in collecting the cases. Yu Zhang and Ai‐Hong Chen helped organize the data. Jie‐Ping Lei helped analyzed the statistical data. Zhen‐Guo Zhai gave support to the study. All authors have given approval to the final version of the manuscript.

## CONFLICT OF INTEREST

The authors declare no conflict of interest.

## ETHICS STATEMENT

The study protocol was approved by China‐Japan Friendship Hospital's Human Research Ethics Committee (2020‐95‐K59). All patients gave informed written consent before inclusion.
